# Functional Relevance of Dual Olfactory Bulbs in Olfactory Coding

**DOI:** 10.1523/ENEURO.0070-21.2021

**Published:** 2021-09-02

**Authors:** Praveen Kuruppath, Li Bai, Leonardo Belluscio

**Affiliations:** Developmental Neural Plasticity Section, National Institute of Neurological Disorders and Stroke, National Institutes of Health, Bethesda, MD 20892

**Keywords:** channelrhodopsin-2, foot shock, light zone, olfactory bulb, safe zone

## Abstract

Bilateral convergence of external stimuli is a common feature of vertebrate sensory systems. This convergence of inputs from the bilateral receptive fields allows higher order sensory perception, such as depth perception in the vertebrate visual system and stimulus localization in the auditory system. The functional role of such bilateral convergence in the olfactory system is unknown. To test whether each olfactory bulb (OB) contributes a separate piece of olfactory information, and whether information from the bilateral OB is integrated, we synchronized the activation of OBs with blue light in mice expressing ChIEF in the olfactory sensory neurons (OSNs) and behaviorally assessed the relevance of dual OBs in olfactory perception. Our findings suggest that each OB contributes separate components of olfactory information, and the mice integrate the bilaterally synchronized olfactory information for olfactory identity.

## Significance Statement

Identifying an odor is the first step in olfactory coding, as it is critical for the survival of most animals. Bilateral olfactory bulbs (OBs) help rodents to localize the odor source and navigate accordingly. However, it is unclear whether the bilateral OBs play a role in determining the identity of an olfactory information. In this study, by the controlled activation of unilateral and bilateral OBs with blue light in tetO-ChIEF-Citrine mouse line, we studied the behavioral responses to the light pulse stimulation in unilateral and bilateral OB. we found that each OB provides distinct olfactory information and rodents integrate information from the two bulbs to identify an olfactory stimulus.

## Introduction

The perception of odor is crucial for the survival of most animals, because it is essential for navigation, finding food sources, and avoiding predators. Convergence of sensory inputs from a bilateral receptive field is a fundamental aspect of the biological sensory systems to extract information about the environment. In the visual, auditory, and somatosensory system, this bilateral convergence is well established for depth perception (Ohzawa et al., 1997; Anzai et al., 2011), sound localization, (King et al., 2001; Konishi, 2003), and object localization (Shuler et al., 2001, 2002). The significance of such bilateral convergence and the functional relevance of dual olfactory bulbs (OBs) in the olfactory system remain unclear.

The identity of an odor, along with its concentration, are two fundamental aspects of olfactory information (Zhou and Belluscio, 2012; Storace and Cohen, 2017; Kepple et al., 2019; Parabucki et al., 2019). Animals move toward increasing odorant concentration to locate food, mates, and avoid predators by moving away following decreasing odorant concentrations. To achieve this effect, animals may rely on a comparison of odorant information sampled through the bilateral symmetric nostrils (Raman et al., 2008; Esquivelzeta Rabell et al., 2017). Previous studies have shown that rodents integrate bilateral cues from the nostrils to localize the odorant sources (Rajan et al., 2006; Catania, 2013). However, it is unknown whether bilateral olfactory inputs are integrated and perceived as a single olfactory information.

In this study, we controlled the sensory inputs to the OBs with blue light in tetO-ChIEF-Citrine mice line, where channelrhodopsin-2 is expressed in all the olfactory sensory neurons (OSNs) and studied the behavioral responses to the light pulse stimulation. We found that each OB provides separate olfactory information, and that the perception of an olfactory stimulus reflects the composite of information provided by the two OBs.

## Materials and Methods

### Experimental animals

All animal procedures conformed to National Institutes of Health guidelines. Mice were bred in-house and were maintained on a 12/12 h light/dark cycle with food and water *ad libitum*.

The tetO-ChIEF-Citrine line, was generated from pCAGGS-I-oChIEF- mCitrine-I-WPRE (7.7 kb; Roger Tsien, University of California, San Diego), which contains the coding sequence for mammalian-optimized ChIEF fused to the yellow fluorescent protein Citrine, at the National Institute of Mental Health Transgenic Core Facility (Bethesda, MD) as previously described (Lin et al., 2009; Cheetham et al., 2016). The OMP-tTA knock-in mouse line expressing the tetracycline transactivator protein (TTA) under the control of the OMP-promoter was a gift from Joseph Gagos. Experimental animals were OMP-tTA+/−/tetO-ChIEF-Citrine+/− (OMP-ChIEF), generated by crossing heterozygous tetO-ChIEF-Citrine (tetO-ChIEF-Citrine+/−) with homozygous OMP-tTA mice (OMP-tTA−/−).

### Genotyping

OMP-ChIEF pups were identified by the visualization of fluorescence in the nose and OB of P0–P2 (postnatal day0 - postnatal day2) pups under epifluorescence illumination.

### Animal preparations

Data were collected from 12 OMP-ChIEF mice (eight males and four females) and four male wild-type mice. Experimental animals were prepared as described previously (Sparta et al., 2011; Ung and Arenkiel, 2012). Briefly, mice were anesthetized with an intraperitoneal injection of ketamine/xylazine mixture 100 and 10 mg/kg body weight, respectively. Each animal was fixed with a stereotactic frame with the head held in place by a bar tie to each temporal side of the skull. The animals were kept warm with hand warmers (Grabber). Surgery was started when the animal showed no movement in response to foot pinching. A craniotomy was performed above the skull over each OB. Fiber optic pins were implanted on the dorsal surface of each OB (4.3 mm anterior from bregma), as described previously (Sparta et al., 2011; Ung and Arenkiel, 2012; Li et al., 2014). Mice were injected with ketoprophen (5 mg/kg) immediately after the surgery. Animals were allowed to recover in their home cage for one week.

### Behavioral procedure and training

#### Light stimulation and foot shock avoidance training

**Figure 1.**
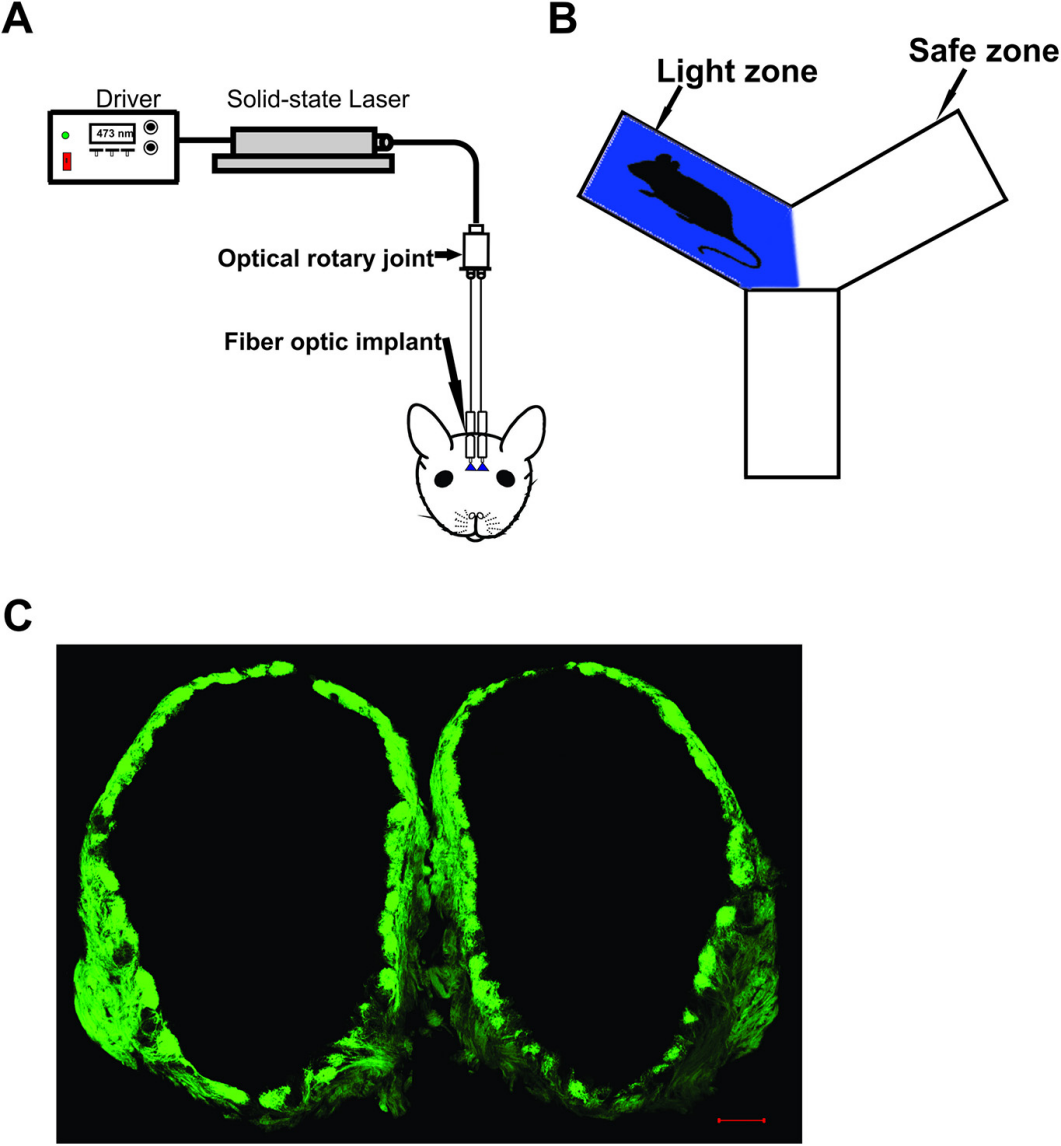
Optogenetic stimulation of OB. ***A***, Schematic diagram of the functional system. ***B***, Behavioral setup. Light zone indicates the area where the light stimulation is delivered. Safe zone indicates the area where mice can escape from the light stimulation and foot shock. ***C***, Fluorescence image of the OB in an OMP-ChIEF mouse. Green indicates the expression of ChIEF tagged with a green fluorescent protein (ChIEF-GFP). Scale bar: 100 μm.

Behavioral training began after the animals recovered from the surgery (one week). Training was performed for 2 d on a “two-arms maze,” modified from “Y” maze, with two equally sized open arms and one permanently closed arm. Each open arm was independently paved with an electric grid shock floor. The mice were connected to a 400-μm core-diameter optical fiber attached to a 473-nm solid-state variable-power laser (LaserGlow Technologies; Fig. 1*A*) and allowed to habituate in the two-arms maze, for 15 min. The time spent in each arm of the two-arms maze, was recorded and assessed for arm preference. The preferred arm for the mice was selected as the light zone and the opposite arm served as the safe zone. If the mice did not demonstrate a preference for either arm, the light zone was randomly selected. After the habituation, mice were re-introduced to the two-arms maze, for the 10-min training session. A 10-min reinforcement training was performed every day 60 min before the test session to enhance learning. The training included light stimulation followed by a mild foot shock. The foot shock avoidance training is paired with either left or right OB stimulation. The light stimulation and the foot shock were delivered in the light zone when the mice completely entered that zone (Fig. 1*B*). The mice had free access to the safe zone to escape the foot shock. Light stimulation, consisting of a train of ten light pulses of 50-ms duration with an interval of 150 ms, was externally triggered by a Master-8 timer (A.M.P.I.; Li et al., 2014; Erofeev et al., 2019; Quinlan et al., 2019). The output power of the light pulses was measured and adjusted to 20–22 mw with reference to previous studies (Lin, 2011, 2012; Sparta et al., 2011). The mild foot shock (0.65 mA, 5 s) generated by a stand-alone shock generator (Med Associates) was delivered 2 s after the light stimulation by Master-8 timer. The mice were trained to move to the safe zone when the light stimuli and the foot shock were delivered in the light zone.

#### Light zone avoidance test

The light zone avoidance test was performed for 2 d. A reinforcement training was performed each day before the testing. The testing began 60 min after the reinforcement training. Before the testing, the electric grid shock floor was removed from the two-arms maze, so the mice did not experience foot shock during the test session. The activity of the mice in the two-arms maze, was evaluated in blocks of three trials and each trial was 15 min. In the first trial, the mice were allowed to explore the arena without any light stimulation and assessed the baseline behavior. The time spent in each arm was calculated to evaluate arm preferences after the foot shock training session. For the following trials, the light zone and safe zone were selected, as indicated previously. The time spent in each arm was calculated by tracking the animal’s movement in the two-arms maze, using Any-maze video tracking software (Stoelting). A heatmap was also generated for each trial which displays the amount of time mice spent in different parts of the arena. A range of colors indicate the total time spent in the area with blue indicating the shortest and the red as the longest time.

#### Perfusion and immunohistochemistry

At the end of behavioral testing, the mice were anesthetized with 200 mg/kg ketamine and 20 mg/kg xylazine and transcardially perfused with ice-cold PBS followed by 4% paraformaldehyde (PFA). OB were dissected out and embedded in 10% gelatin and postfixed/cryopreserved in 15% sucrose and 2% PFA in PBS overnight at 4°C, cryopreserved in 30% sucrose in PBS for 24 h at 4°C and flash frozen in 2-methyl butane on dry ice. Coronal sections were cut using a cryostat (Leica Microsystems) at 50 μm and stored at −80°C.

For immunohistochemistry, free-floating sections were incubated for 20 min in 1% sodium borohydride in TBS, blocked in the blocking medium containing 5% horse serum, 0.1% gelatin and 0.5% Triton X-100 for 1 h, and incubated with OMP primary antibody (goat, Wako, 1:1000) in 3% horse serum and 0.2% Triton X-100 for 24 h at 4°C, then donkey-anti-goat Cy3 secondary antibody (Jackson ImmunoResearch, 1:600) for 2 h at room temperature. Sections were mounted in Vectashield mounting medium (Vector Laboratories). Images were acquired using a Zeiss LSM 510 laser scanning confocal microscope.

### Statistical analysis

All statistical analyses were done by GraphPad Prism software (GraphPad). Statistics are displayed as mean ± SEM. Paired *t* test was used for the comparison. Differences were determined significant when *p* < 0.05.

## Results

### Each OB provides distinct olfactory information

**Figure 2.**
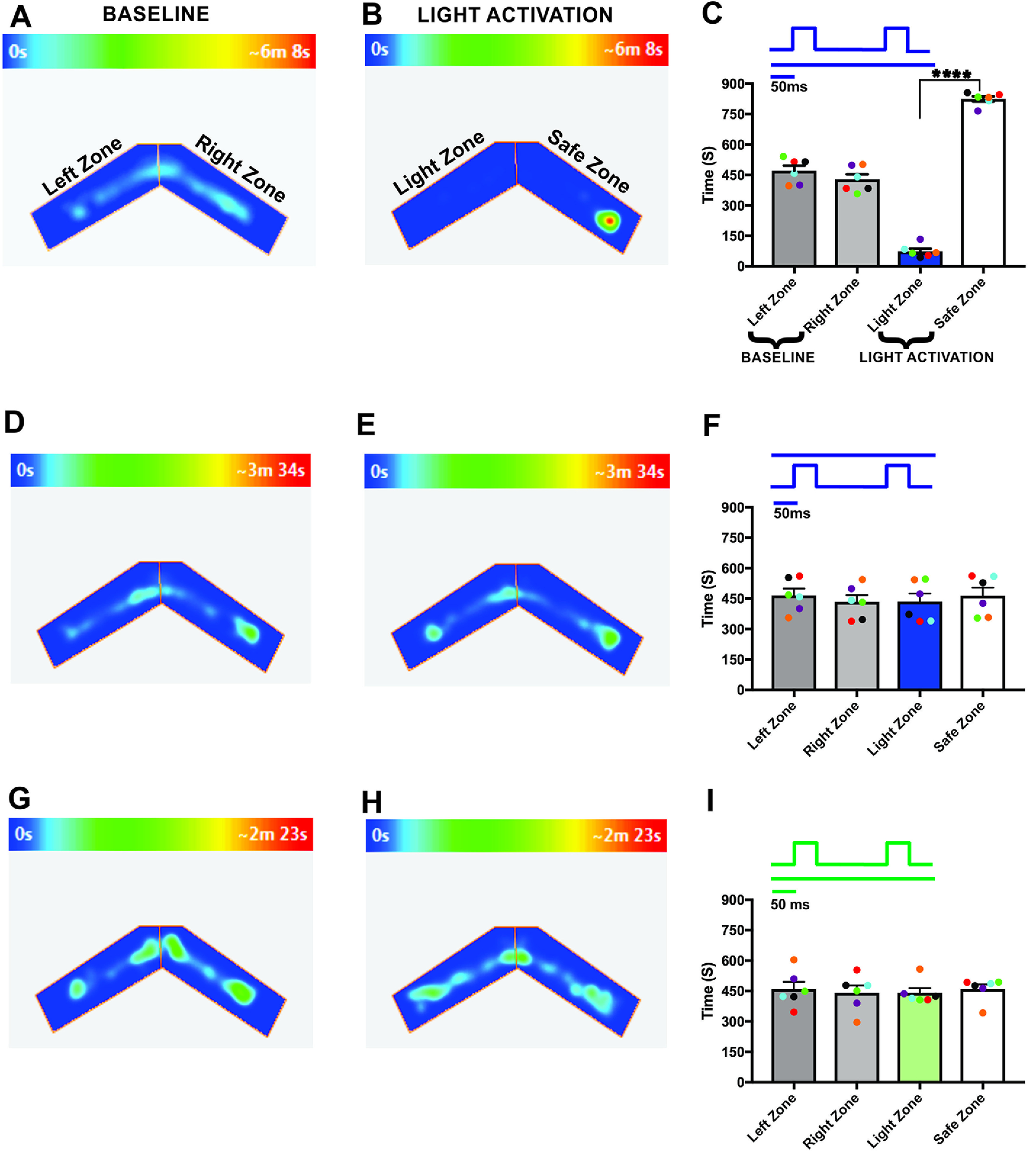
Distinct odor information from individual OB. ***A***, ***B***, Example of heat-map showing animal’s position in two-arms maze, during baseline (***A***) and ipsilateral OB stimulation (***B***). ***C***, Average amount of time explored in each zone in baseline (left zone, right zone) and ipsilateral OB stimulation trials (light zone, safe zone). ***D***, ***E***, Heat-map of mouse position during baseline (***D***) and contralateral OB stimulation (***E***). ***F***, Average amount of time spent in each zone in baseline and contralateral OB stimulation trials. ***G***, ***H***, Heat-map of mouse position during baseline (***G***) and ipsilateral green light stimulation (***H***). ***I***, Average amount of time spent in each zone in ipsilateral green light stimulation (∗∗∗∗*p* < 0.0001, *n* = 6 animals).

Previous studies have shown that odor information is projected unilaterally (Marrone, 2013) in the vertebrate olfactory system, indicating that odors may be encoded separately in each hemisphere. To investigate the unilateral coding of olfactory information, we optically stimulated ChIEF expressing OSNs in each OB (Fig. 1*C*) and assessed the behavioral response to light stimulation. Here, we performed foot shock avoidance training paired with either left or right OB stimulation. To avoid the activation of same glomeruli in each OB, we implanted the optical fiber in different locations in each OB. Therefore, the light stimulation on each OB activates a different set of glomeruli, eliciting different olfactory information in each OB. After training, we tested the light zone avoidance response to both ipsilateral and contralateral OB stimulation. First, as a control, we calculated the time spent in each arm by allowing the mice to freely explore the arena in the absence of stimulation. The aim of the baseline behavior analysis was to determine whether the mice have any preference to a particular arm of the two-arms maze, after the foot shock training. Our baseline data show that mice spent equal amounts of time in both arms of the maze (left zone, 471.6 ± 25.57 s, right zone, 428.4 ± 25.57 s, *p* = 0.44; Fig. 2*A*). Then, we delivered the light stimulation to ipsilateral OB. We found that, mice avoided the light zone during the ipsilateral OB stimulation and spent most of their time in the safe zone (light zone, 74.67 ± 13.07 s; safe zone, 825.3 ± 13.07 s, *p* ≤ 0.0001; Fig. 2*B*,*C*; Movie 1, *n* = 6). We also tested whether the avoidance response to the light stimulation was equally probable in both arms of the maze by delivering the stimulation when the mice reached the safe zone. We found that mice avoided the safe zone during the ipsilateral light stimulation, indicating that the avoidance response is clearly linked to the olfactory information, but not to spatial information. Since we did not see a difference in the avoidance response, we pooled the data from both stimulus groups.

Movie 1. Unilateral foot shock trained mice avoid light zone during ipsilateral OB stimulation. 10.1523/ENEURO.0070-21.2021.video.1

Next, we performed light stimulation on the contralateral OB. Our results show that the time spent in each arm during the baseline behavior trial (left zone, 466.4 ± 33.25 s, right zone, 433.6 ± 33.25 s, *p* = 0.64; Fig. 2*D*) and the light activation trial (light zone, 435.3 ± 39.93 s, safe zone, 464.7 ± 39.93 s, *p* = 0.73; Fig. 2*E*) were similar, indicating that the mice did not identify the foot-shock linked olfactory information. Therefore, they did not avoid the light zone during contralateral OB stimulation (Fig. 2*F*; Movie 2). This suggests that each OB can deliver separate light-induced olfactory information that can be discriminated by the mice.

Movie 2. Unilateral foot shock trained mice did not avoid light zone during contralateral OB stimulation. 10.1523/ENEURO.0070-21.2021.video.2

**Figure 3.**
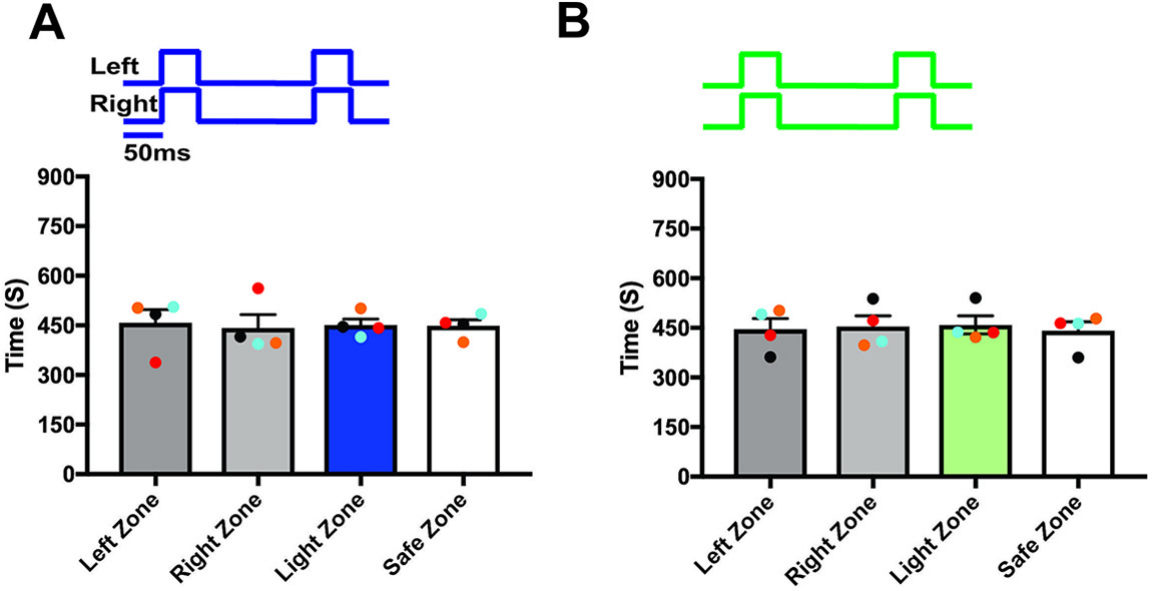
Light stimulation did not activate the olfactory system of the wild-type mice. ***A***, Average amount of time spent in each zone in baseline and experiment trials during blue light stimulation. ***B***, Average amount of time spent in each zone in baseline and experiment trials during green light stimulation (*n* = 4).

To verify that the observed responses from the light stimulation were the result of activation of the ChIEF-expressing neurons and not from the use of light as a visual cue, we used green light (540 nm, output power, 20–22 mw), which does not activate ChIEF. Mice are much less sensitive to such long wavelength light (Denman et al., 2017, 2018; Peirson et al., 2018). During the green light stimulation, we did not observe significant behavioral difference from the baseline behavior (left zone, 459 ± 36.13 s and right zone, 441 ± 36.13 s, *p* = 0.81; light zone, 441 ± 23.88 s and safe zone, 459 ± 23.88 s, *p* = 0.72; Fig. 2*G–I*), confirming that the mice did not use visual cues to perform the task. We also performed the test with wild-type mice and confirmed that visual cues were not involved in solving the behavioral task [left zone, 457.8 ± 40.21 s and right zone, 442.3 ± 40.21 s, *p* = 0.86; light zone, 451 ± 18.03 s and safe zone, 449 ± 18.03 s, *p* = 0.96 (Fig. 3*A*); left zone, 445.8 ± 32.33 s and right zone, 454.3 ± 32.33 s, *p* = 0.90; light zone, 458.8 ± 27.30 s and safe zone, 441.3 ± 27.30 s, *p* = 0.77 (Fig. 3*B*, *n* = 4)]. These results indicate that OMP-ChIEF mice were using only the light activation of the olfactory system as the cue to solve the behavioral task, rather than light detection via other modalities.

### Bilateral integration of olfactory information

**Figure 4.**
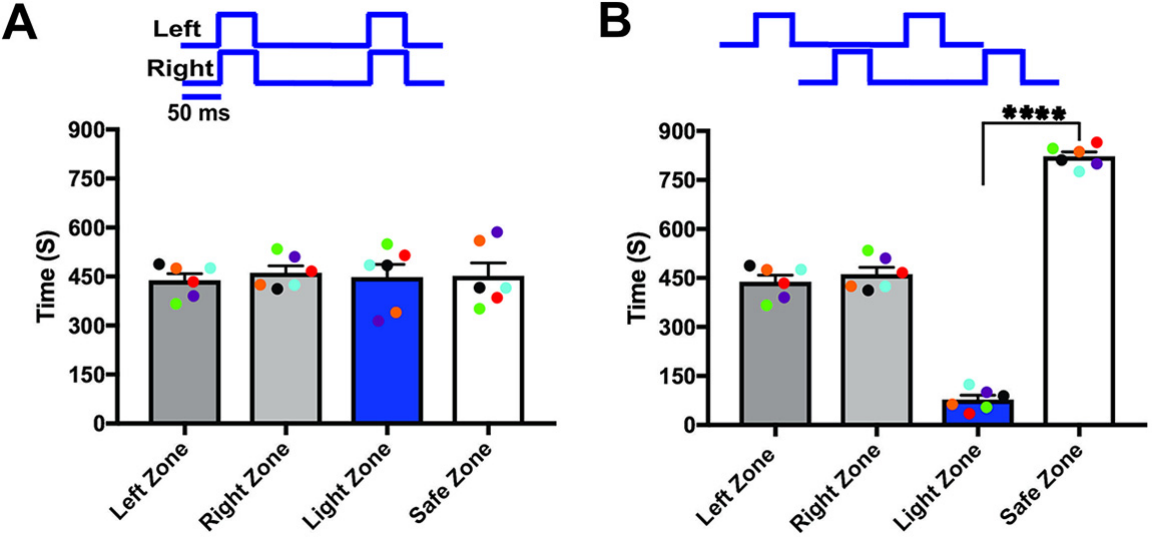
Synchronized bilateral OB stimulation alters the unilateral odor identity. ***A***, ***B***, Mean time explored during the synchronous and asynchronous OB stimulation to the unilaterally trained mice (∗∗∗∗*p* < 0.0001, *n* = 6 animals).

Previous tracer injection studies have shown that olfactory information is exchanged to the contralateral OB through anterior olfactory nucleus pars externa (AONpE; Yan et al., 2008). However, it is unknown whether the olfactory information from both OBs is integrated in odor perception. To assess whether the bilateral olfactory information is integrated in olfactory perception, we delivered synchronized light stimulation to both OBs of unilaterally trained mice and observed if the mice can distinguish the foot-shock linked olfactory stimulus from synchronized dual bulb stimulation. We found that mice visited both arms for similar amounts of time during the baseline behavior trials versus trials in which the two OBs were synchronously stimulated (left zone, 438.2 ± 20.67 s, right zone, 461.8 ± 20.67 s, *p* = 0.59; light zone, 447.8 ± 39.57 s, safe zone, 452.2 ± 39.57 s, *p* = 0.95; Fig. 4*A*; Movie 3). This implies that mice detected the synchronized bilateral olfactory stimulus differently from the unilateral olfactory stimulus linked to the foot-shock. Thus, mice perceive the combined olfactory information received from the ipsilateral and contralateral OBs during synchronized bilateral OB stimulation differently from the information received from each bulb.

Movie 3. Unilateral foot shock trained mice did not avoid light zone during synchronized bilateral OB stimulation. 10.1523/ENEURO.0070-21.2021.video.3

To verify that there is bilateral integration, we delivered the light stimulation asynchronously with a 50-ms delay between ipsilateral and contralateral OB stimulation. This protocol assumed that asynchronous OB stimulation did not allow the mice to integrate the olfactory information because each OB receives the light stimulation at a different time. We observed that when the OBs were stimulated asynchronously, mice identified a distinct light-driven olfactory stimulus paired with the foot-shock and avoided the light zone (light zone, 77.58 ± 13.36 s; safe zone, 822.4 ± 13.36 s, *p* ≤ 0.0001; Fig. 4*B*; Movie 4). We also evaluated the difference in the avoidance response when the contralateral OB received light stimulation before the ipsilateral OB. This was done by, in some of the trials, first stimulating the contralateral OB before the ipsilateral OB and examining whether the mice continued to avoid the light zone. We found that mice were able to identify the olfactory stimulus paired with the foot-shock and avoided the light zone. Since we did not see a difference in the avoidance response, we pooled the data from both stimulus groups. Together, these results suggest that during asynchronous stimulation, olfactory information from the two OBs is processed separately.

Movie 4. Unilateral foot shock trained mice avoid light zone during asynchronous bilateral OB stimulation. 10.1523/ENEURO.0070-21.2021.video.4

### Unified perception of bilateral olfactory inputs

**Figure 5.**
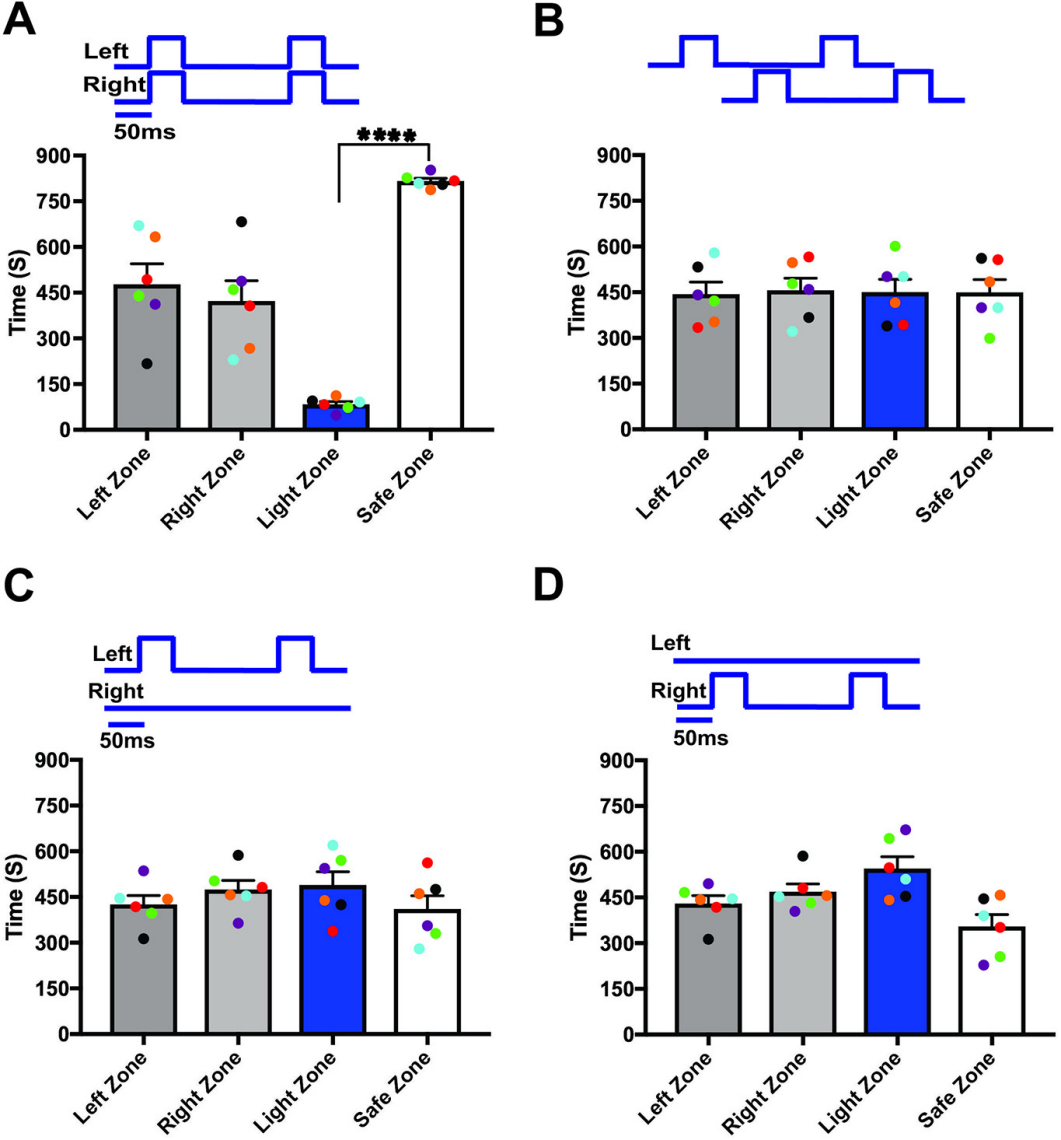
Unified perception of synchronized bilateral olfactory inputs. ***A***, ***B***, Mean time explored during the synchronized and asynchronized bilateral OB stimulation to the bilaterally trained mice. ***C***, ***D***, Mean time explored during unilateral light stimulation (∗∗∗∗*p* < 0.0001, *n* = 6 animals).

Sensory information from bilateral receptive fields is combined into a single, unified percept in most sensory systems. However, the presence of a unified perception in the vertebrate olfactory system is unknown. To determine the unified perception of bilateral olfactory stimulation, we performed light stimulation and foot-shock avoidance training on a new group of animals (*n* = 6). Here, we delivered light stimulation to both OBs simultaneously and paired it with foot shock. After the training, we tested the response of mice in the light zone avoidance test during synchronized bilateral OB stimulation. Our results show that when both OBs were synchronously stimulated, mice avoided the light zone, indicating that mice can identify the olfactory information linked to the foot-shock (light zone, 83.67 ± 8.891 s; safe zone, 816.3 ± 8.891 s, *p* ≤ 0.0001; Fig. 5*A*; Movie 5, *n* = 6). We then tested whether the mice identify the foot-shock linked olfactory stimulus when the two OBs are stimulated asynchronously. This protocol assumes that if the mice relied on the olfactory information from the left or right OB to avoid the light zone, mice would identify the foot-shock linked olfactory stimulus and avoid the light zone. In contrast to this prediction, we found that the mice did not identify the olfactory information linked with foot-shock and continued to stay in the light zone (light zone, 450.2 ± 42 s; safe zone, 449.8 ± 42 s, *p* = 0.99; Fig. 5*B*; Movie 6). Our results suggest that the mice integrate the olfactory information from both OBs during synchronized stimulation and perceive it as one olfactory stimulus. However, during the asynchronized stimulation, mice perceived two independent streams of information.

Movie 5. Bilateral foot shock trained mice avoid light zone during synchronized bilateral OB stimulation. 10.1523/ENEURO.0070-21.2021.video.5

Movie 6. Bilateral foot shock trained mice did not avoid light zone during asynchronous bilateral OB stimulation. 10.1523/ENEURO.0070-21.2021.video.6

Next, we tested whether the mice can identify the foot-shock linked bilateral olfactory stimulus from single OB stimulation. We independently stimulated the left and right OBs of the bilaterally trained mice. We found that during the independent activation of each OB, mice did not avoid the light zone, but spent equal or additional time in the light zone as in baseline behavior trials [left zone, 425.5 ± 29.7 s and right zone, 474.5 ± 29.7 s, *p* = 0.45; light zone, 489.3 ± 43.26 s and safe zone, 410.7 ± 43.26 s, *p* = 0.40 (Fig. 5*C*); left zone, 430.3 ± 25.72 s and right zone, 469.7 ± 25.72 s, *p* = 0.48; light zone, 545 ± 39.18 s and safe zone, 355 ± 39.18 s, *p* = 0.06 (Fig. 5*D*)]. This suggests that unilateral stimulation of each OB provides only a portion of the information delivered by bilateral olfactory stimulation. The result confirms our previous hypothesis that the two OB provide two separate pieces of information, which are encoded independently in each hemisphere. Furthermore, our results demonstrate that each OB provide distinct olfactory information, which can be combined and perceived as a single olfactory stimulus.

**Figure 6.**
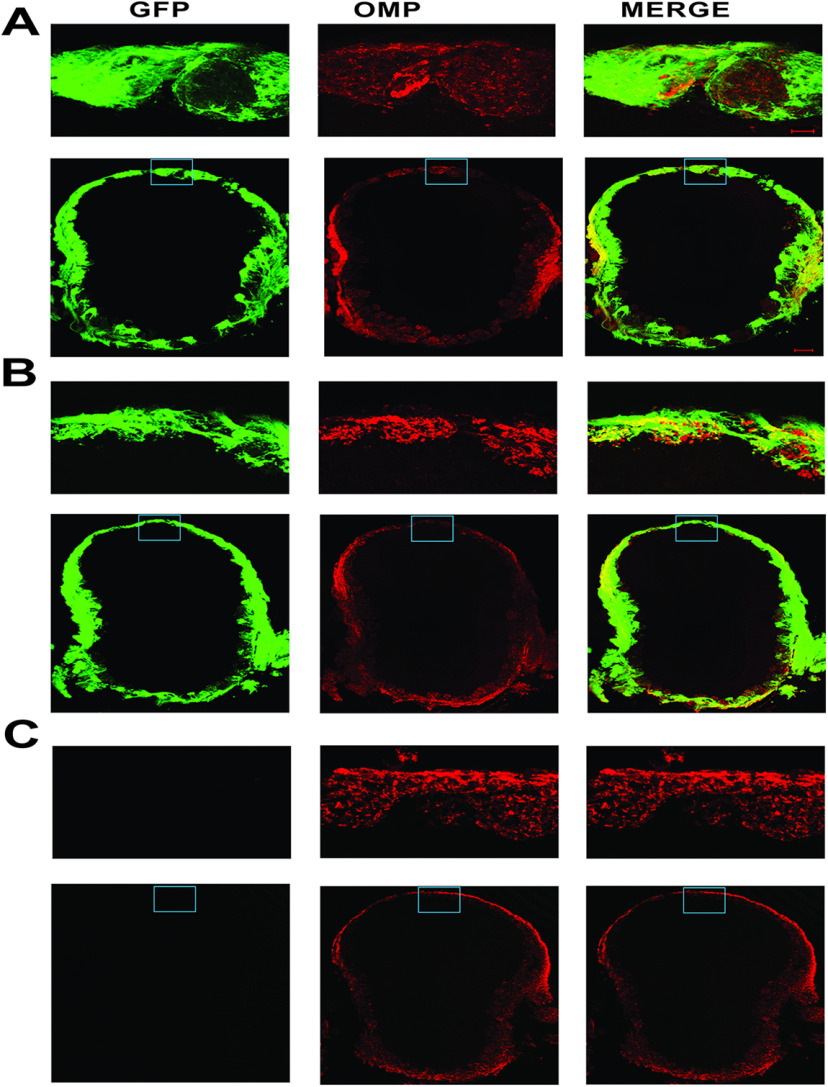
ChIEF expression in OMP (Olfactory marker protein) positive neurons. ***A***, ChIEF expression in OMP positive OSNs in 12-week-old OMP-ChIEF control animal. ***B***, ChIEF expression in OMP positive OSNs in 12-week-old OMP-ChIEF experimental animal. ***C***, No ChIEF expression on the OMP positive neurons of 12-week-old wild-type animal. Scale bars: 100 μm (***A–C***) and 20 μm (inset).

Finally, we visually compared the OMP positive OSNs in 12-week-old OMP-ChIEF control, experimental and wild-type mice and examined whether light stimulation induced a reduction in ChIEF expression and degeneration of OMP positive neurons in the wild-type and experimental mice. Our immunohistochemistry results show that light stimulation did not induce a reduction in the ChIEF expression or degeneration of the OMP positive OSNs (Fig. 6*A–C*).

## Discussion

Several studies have shown that rodents use bilateral cues to localize odor source for navigation, finding food sources, and avoiding danger. Balanced and lateralized sensory inputs are the key strategies typically involved in bilateral localization. Recent studies in rodents show that odor discrimination require lateralization of the olfactory information, demonstrating the significance of lateralized sensory inputs in odor discrimination (Cohen et al., 2015; Cohen and Wilson, 2017). A previous study in humans by Thuerauf and colleagues also demonstrated the significance of lateralization for the perception of odor intensity and hedonic evaluation (pleasantness/unpleasantness; Thuerauf et al., 2008). Their study show that olfactory intensity estimates represent the most sensitive parameter of olfactory lateralization, suggesting that olfactory laterality is mainly based on intensity rather than identity.

While these studies report the significance of bilateral sampling for accurate odor source localization (Porter et al., 2005; Rajan et al., 2006; Louis et al., 2008; Catania, 2013; Esquivelzeta Rabell et al., 2017), none of the studies examined the significance of bilateral integration of olfactory information for identifying an odor. Taking advantage of the transgenic mice, our study showed that rodents combine the synchronously sampled olfactory information provided by the two OBs into a single olfactory percept. The integration of such bilateral olfactory information is likely to play a central role in the exchange of olfactory information and the formation of a unified odor percept may help to generate behaviorally relevant information regarding the animal’s surrounding environment.

A previous study reported that olfactory information is projected unilaterally (Shepherd, 2004), suggesting that odorant information is encoded separately in each hemisphere. Consistent with this, our study provides evidence for distinct olfactory information processing in each hemisphere. We show that, when stimulated independently, the ipsilateral and contralateral OBS provide unique olfactory information, and mice can clearly identify the olfactory information from two bulbs (Fig. 2*C*,*F*).

In humans, Zhou and Chen (Zhou and Chen, 2009) examined how odors are perceived when each nostril receives a different odor. In their experiment, phenylethyl alcohol (PEA; rose like smell) and n-butanol (marker like smell) were delivered to each nostril simultaneously and the subjects were asked to report whether the odor perceived was “rose” or “marker.” They found that the subjects perceived either the marker or the rose smell. The odor perception was constant within a sniff and could change between sniffs. This suggests that although each hemisphere presumably perceived a different odor, only one percept dominated rather than both contributing equally. Sniffing, flow rate and the complex interactions between odorants and the epithelium may influence this dominance of one percept leading to the perception of one odorant than contributing equally from each OB.

The functional relevance of the dual OBs in olfactory information processing is still an open question. A recent study in *Drosophila*, found that olfactory receptor neurons (ORNs) project bilaterally to both sides of the brain. When an odor activates the antennal lobe asymmetrically, ipsilateral central neurons begin to spike a few milliseconds earlier at a higher rate than the contralateral neurons, allowing the fly to localize the direction of the odor source (Gaudry et al., 2013).

Previous studies in rodents reported that isofunctional glomeruli are interconnected not only within the hemisphere, but also between the hemispheres through AON, creating a mirror symmetric olfactory map in each hemisphere (Schoenfeld and Macrides, 1984; Ressler et al., 1994; Mombaerts et al., 1996; Wilson, 1997; Uchida et al., 2000; Mainland et al., 2002; Grobman et al., 2018; Kermen et al., 2020). Together, these reports suggest that dual OBs enhance the odor source localization and navigation. However, Grobman and coworkers (Grobman et al., 2018) suggest that bilateral projection is not necessary to get a bilateral response difference. Instead, a unilateral projection can result in a strong bilateral response difference. Their study shows that a unilateral projection pathway generates a higher bilateral response difference than the bilateral pathway. Since response strength is correlated with response latency, the time difference in response latency will be higher in unilateral pathway than the bilateral pathway, achieving a better odor localization in unilateral pathway. Their study suggest that the main role of bilateral projection is not odor localization, but the sharing of odor identity information across the hemispheres.

In agreement with this study, we propose that light stimulation of a single OB forms a neural representation of an odor’s molecular identity in both hemispheres. In addition, we propose that the final odor identity is represented not only by the combinatorial activation of specific ORs, as previously reported (Malnic et al., 1999; Saito et al., 2009), but also by the integration of the olfactory information from the contralateral OB. Based on the previous study (Grobman et al., 2018) and our results, we suggest that one of the main roles of dual OB is the coding of odor identity. Further research is required to fully understand how olfactory cortical neurons integrate the dual olfactory information for odor identity and odor localization and the significance of the bilateral integration of olfactory information.

In natural environments, animals confront more complex problems such as, having to identify the quality, and complexity of an odor mixture. The use of suitable physiological and psychophysical paradigms will be a crucial step for further understanding the complexity of neural coding in the olfactory system.

## Synthesis

Reviewing Editor: Christina Zelano, Northwestern University Decisions are customarily a result of the Reviewing Editor and the peer reviewers coming together and discussing their recommendations until a consensus is reached. When revisions are invited, a fact-based synthesis statement explaining their decision and outlining what is needed to prepare a revision will be listed below. The following reviewer(s) agreed to reveal their identity: Diego Restrepo.

While both reviewers agreed that this study has the potential to contribute very interesting and novel insights, several concerns were raised that will need to be addressed before the manuscript can be considered further.

First, there is an important control experiment missing. As is, the behavioral results could potentially be explained by mice perceiving laterality of odorant stimulation rather than odorant identity. Because of this, the conclusion stated in the manuscript is not supported by the data. In discussions, the reviewers agreed that an appropriate control experiment would be to place the fibers in the same place in each bulb, and test whether mice can differentiate bilateral from unilateral stimulation. If this control is run, and the mice cannot differentiate bilateral stimulation from unilateral stimulation, then the authors would be correct that the information conveyed upstream is information on odorant features. If they can still differentiate between these two conditions then the mice could be perceiving information on whether stimulation is lateral. Placing fibers in the same place in both bulbs may be technically challenging, and could potentially not work (though might be more feasible if the fiber is larger). If this control experiment is not feasible, then the conclusions of the paper should be adjusted to include alternate explanations.

Second, the writing in the manuscript needs improvement. The framing, discussion of significance, methods, and previous literature all need improvement.

Third, a number of clarifications on methods and data were requested. Please read below the reviewers detailed comments and provide clarifications in figures and on analyses where requested.

Please see more detailed comments from both reviewers below:

Reviewer 1:

Mammals have bilateral olfactory sensing organs, and recent work has highlighted that they can make use of stereo-odor cues to improve discrimination with respect to odor navigation, but questions remain as to whether they play additional perceptual roles. More specifically, it is unclear whether the brain integrates sensory input to the two hemispheres as single olfactory perceptions.

The authors carry out a relatively simple yet interesting experiment to address this question by asking how a mouse perceives two distinct stimuli delivered to one hemibulb versus another, versus when delivered simultaneously to both hemibulbs. Instead of using odors, the authors use an optogenetic strategy in which a channerhodopsin2 variant is expressed in all olfactory sensory neurons. Optic fibers implanted into both olfactory bulbs allowed for stimulation of one, the other, or both hemibulbs.

To test the animal’s perception of this stimulus they were trained to associate light stimulation with a foot-shock. The animal’s avoidance response was quantified in subsequent experiments in which only light stimulation was delivered.

In the first experiment they demonstrate that mice can discriminate light-shock pairing of one bulb versus another. After pairing light stimulation of the ipsilateral side with a foot shock, ipsilateral stimulation alone evoked an avoidance response, while stimulation of the contralateral side did not. The implication being that mice can differently perceive optical stimulation of one bulb from the other. Next they demonstrated that mice can also discriminate stimulation of the ipsilateral side from bilateral stimulation (which of course includes the ipsilateral side stimulation). A reversal of the experiment was also carried out where bilateral stimulation was paired with a foot shock, and the mice responded to bilateral stimulation with avoidance behavior, but not ipsilateral alone, contralateral alone, or a sequence of rapidly alternating ipsi/contralateral stimulation. In other words, the mice appear to perceive bilateral stimulation as an entirely different perception than ipsilateral stimulation alone.

Is bilateral stimulation analogous to an odor mixture - or do the authors view it more as a completely different odor that evokes subsets of the same glomeruli? If a mixture, many odor pairings create perceptually distinct mixtures. However, the fact that they blend across hemispheres is perhaps, the point of this paper. Despite the output of the bulb being primarily ipsilateral, the brain perceives stimulation across the two bulbs as being a single odor object as opposed to two different objects.

Overall, this experiment was interesting and carefully done. Several important control experiments were carried out to control for possible heat and visual cues in wildtype mice. However, the manuscript is poorly written. The significance and methods are poorly explained and the past literature is poorly summarized. I hesitate to make a decision on this first draft because I think the work that was done would greatly benefit from a better framing and I am very curious to read it again in an improved context.

Some minor comments/questions below.

Can the authors comment on whether this experiment could have been similarly done by simply presenting two different odors to the mouse’s nostrils - either to one side, the other or together? Presumably this would be very difficult to carry out in a controlled fashion and I’m assuming this is the strongest reason for using ChIEF?

The baseline behavior measured following training showed that the mice had no spatial preference in the modified maze. Since mice are given foot shocks in the “light” zone, wouldn’t they learn to associate that side with foot shock which should have evoked a natural fear response? After reading it several times I’m guessing this was intentionally made to be ambiguous to the mouse but I don’t think it is explained and should be clarified.

I was surprised that the mice exhibited no generalization. If suddenly a completely novel odor perception predicted a foot shock, you might be on the alert for all completely novel and surprising odor experiences. That said, it’s impossible to know what perception optical stimulation of a large part of the dorsal bulb would evoke, but it does seem likely that it is something unique from anything the animal had ever smelled before.

The histology analysis is confusing and doesn’t seem to have any rationale associated with it. Is there a reason to think that this analysis was necessary? If so clarify. Moreover, the description of those results (i.e., lines 265-271) are repetitive and lack detail.

For example, “our IHC results show that light stimulation did not induce reduction in the ChR2 expression” suggests that they compared the fluorescence from mice that had undergone optical stimulation from those that had not. Presumably there was some kind of criteria to make these comparisons (even if it was just a simple “visual inspection”) but this is not described.

I don’t understand the number in the heat map. 2A indicates a total of 368 seconds, but this doesn’t correspond to any of the numbers in panel C. The methods indicate that each trial was 15 minutes so wouldn’t the time spent in these panels be out of at least 15 minutes (if the trials were averaged)? It is hard to imagine how the colors in 2A would add up to 15 minutes -- it could just be a trick of the color scale or that I’m not understanding but either way it would be helpful to clarify.

The diagrams in Figure 2C, F should be labeled (as well as on the other figures).

The total number of trainings that animals underwent is vaguely described and should be clarified.

Line 181: There is a period between “figure” and “3"

Line 213: I think its probably wrong to describe the experience of OB optical stimulation as “smell”. Perceive is probably a more accurate description of what is happening.

Line 221-222: Poorly constructed sentence.

Line 230: No period after “4"

Line 243: “tested to determine"

Line 250: “as a single olfactory information” - I understand what the authors are trying to say here but it reads somewhat awkwardly.

Line 291: I do not understand the sentence: “Here, we report that rodents combine the synchronously sampled bilateral olfactory information for odor identity”.

Line 309: What is a “solid bilateral response difference"

Line 311: I think “and the” should read “and that” - but what does it mean that odor localization is easier in the unilateral projection pathway than the bilateral pathway (i.e., what would easier localization be??)

Line 318: “based on the previous studies", but only one study is cited.

Line 274: No period after Figure 5.

Reviewer 2:

This manuscript addresses the question whether integration of the information received by the two olfactory bulbs contributes to identification of odor identity. The authors asess the association of foot shock with optogenetic stimulation of olfactory bulb input in either bulb or both bulbs. Stimulation is performed on purpose in different dorsal locations in the two bulbs to mimic activation of glomeruli in each bulb. The authors show that the mice distinguish between bilateral simultaneous stimulation and unilateral stimulation or bilateral stimulation that does not overlap over time. They conclude that the information from each bulb is integrated to identify an “olfactory stimulus” made up of information on different features contributed by each bulb. This is in principle an interesting observation, but the data do not convince me of the conclusion.

1. The major problem I find is that the behavioral results could be explained by mice perceiving laterality of odorant stimulation. If this is the case the mice could differentiate whether nostrils are stimulated bilaterally vs unilaterally, and which nostril is stimulated unilaterally. In this case the observations would not have anything to do with perception of “odorant identity”.

2. Let’s assume that the conclusion that the two olfactory bulbs contribute information on odorant features is correct. If this is the case I would question the statement that “mice integrate the olfactory information from each olfactory bulb to identify an olfactory stimulus.” (lines 13-14). In a natural environment this would happen when two different odors enter each nostril separately. This can happen if two different odorants are released from different sites and reach each nostril separately.

3. The fact that the optical fiber is implanted in different dorsal locations in each bulb is only mentioned briefly in line 158. Please give a more detailed description of the location of the fiber. Was a control run where the fibers were placed in the same location bilaterally? If this control were run and the mice cannot differentiate bilateral stimulation from unilateral stimulation then the authors would be correct that the information conveyed upstream is information on odorant features. If they can still differentiate between these two conditions then the mice are perceiving information on whether stimulation is lateral.

4. In line 170-172 there is a brief mention to the fact that a control was run to exclude avoidance of the stimulation because of spatial information. However, the data are not shown. This is an important control. Please expand and show the data.

Minor comments

1. Many of the references are missing the volume and pages.

2. In some places the opsin is called ChR2 (e.g. line 155) and in others it is called ChIEF. These are two different opsins . Please use “ChIEF” throughout the manuscript.

3. Line 163 change “to confirm if” to “to determine whether “.

4. Line 164 delete “almost”.
